# C3c deposition predicts worse renal outcomes in patients with biopsy‐proven diabetic kidney disease in type 2 diabetes mellitus

**DOI:** 10.1111/1753-0407.13264

**Published:** 2022-03-24

**Authors:** Meng‐Rui Li, Zi‐Jun Sun, Dong‐Yuan Chang, Xiao‐Juan Yu, Su‐Xia Wang, Min Chen, Ming‐Hui Zhao

**Affiliations:** ^1^ Renal Division, Department of Medicine Peking University First Hospital Beijing China; ^2^ Institute of Nephrology Peking University Beijing China; ^3^ Key Laboratory of Renal Disease Ministry of Health of China Beijing China; ^4^ Research Units of Diagnosis and Treatment of Immune‐mediated Kidney Diseases Chinese Academy of Medical Sciences Beijing China

**Keywords:** C3, complement, diabetic kidney disease, prognosis, renal pathology, C3, 补体, 糖尿病肾病, 预后, 肾脏病理

## Abstract

**Background:**

Although extensive efforts have been paid to identify reliable predictors for renal outcomes of diabetic kidney disease (DKD) patients in type 2 diabetes mellitus (T2DM), there are still only a limited number of predictive factors for DKD progression. Increasing evidence reported the role of the overactivated complement system in the pathogenesis of DKD. Whether renal complement depositions are associated with renal outcomes of DKD in T2DM is of interest.

**Methods:**

A total of 213 biopsy‐proven DKD patients with T2DM were retrospectively recruited. Clinical and pathological data of the patients were analyzed. Kaplan‐Meier analysis and Cox regression analysis were performed to explore predictors of end‐stage renal disease (ESRD).

**Results:**

During a median follow‐up of 23.0 (12.0, 39.0) months, 100/213 (46.9%) patients progressed to ESRD. C3c and C1q deposition were observed in 133/213 (62.4%) and 45/213 (21.1%) patients, respectively. Kaplan‐Meier analysis revealed patients with C3c or C1q deposition had significantly worse renal outcomes compared with those without C3c or C1q deposition (*p* = .001 and *p* < .001, respectively). Univariate and multivariate Cox regression analysis demonstrated proteinuria (per 1 g/24 h increase, hazard ratio [HR] 1.134, 95% confidence interval [CI] [1.079, 1.191], *p* < .001), interstitial fibrosis and tubular atrophy score (score 2 and 3 vs. 0 and 1, HR 3.925, 95% CI [1.855, 8.304], *p* < .001), and C3c deposition (per 1+ increase, HR 1.299, 95% CI [1.073, 1.573], *p* = .007) were independent predictors for ESRD in DKD patients with T2DM.

**Conclusions:**

C3c deposition in the kidney was associated with worse renal outcomes and was an independent predictor for ESRD in DKD patients with T2DM.

## Graphical abstract

**Highlights**

- C3c deposition in the kidney was associated with worse renal outcomes and was an independent predictor for ESRD in DKD patients with T2DM.
- C3c deposition in the kidney might be a novel biomarker indicating DKD progression in T2DM.

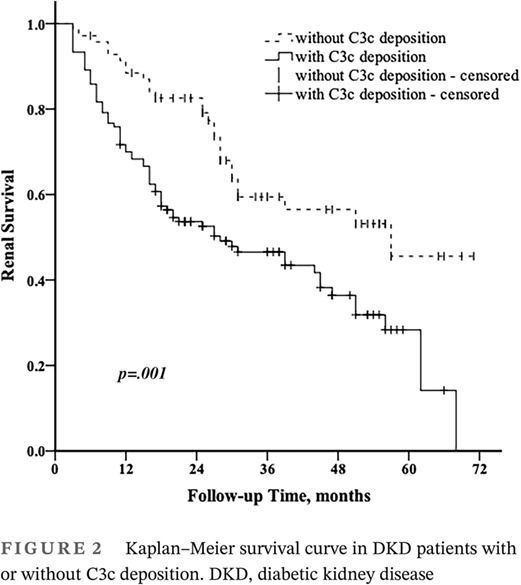

## 摘要

**背景:**

尽管已经付出了大量努力来确定糖尿病肾病(DKD)合并2型糖尿病(T2DM)患者肾脏结局的可靠预测因子, 但DKD进展的预测因子仍然有限。越来越多的证据表明, 过度激活的补体系统在DKD的发病机制中起着重要作用。T2DM患者肾脏补体沉积是否与DKD的肾脏预后有关值得关注。

**方法:**

回顾分析213例经手术病理证实的DKD合并T2DM患者的临床资料。对患者的临床和病理资料进行分析。采用Kaplan‐Meier分析和Cox回归分析探讨终末期肾病(ESRD)的预测因子。

**结果:**

中位随访23.0(12.0, 39.0)个月, 100/213(46.9%)患者进展为ESRD。C3c和C1q沉积分别为62.4%(133/213)和21.1%(45/213)。Kaplan‐Meier分析显示, C3c或C1q沉积的患者与无C3c或C1q沉积的患者相比, 肾脏结局明显差(分别为p=0.001和p<0.001)。单因素和多因素COX回归分析显示, 蛋白尿(每增加1g/24小时, HR 1.134, 95%CI[1.079, 1.191], p<0.001), 间质纤维化和肾小管萎缩积分(2和3分 vs. 0和1分, HR 3.925, 95%CI[1.855, 8.304], p<0.001)和C3c沉积(每增加1+, HR 1.299, 95%CI[1.073, 1.573], p=0.007)是DKD合并T2DM患者ESRD的独立预测因子。

**结论:**

肾内C3c沉积与肾脏结局不良相关, 是DKD合并T2DM患者ESRD的独立预测因子。

## 1 INTRODUCTION

The global prevalence of diabetes mellitus (DM) increased rapidly over the last decades, especially type 2 diabetes mellitus (T2DM).^1^ Diabetic kidney disease (DKD) occurred in 20–40% of patients with DM^2^, ^3^, ^4^ and has become the leading cause of end‐stage renal disease (ESRD).^2^, ^5^ In China, DKD has replaced glomerulonephritis and became the most common cause of chronic kidney disease both in the general population and hospitalized urban population.^6^ Despite extensive efforts to identify reliable biomarkers for renal outcomes, there are still only a limited number of valuable predictive factors for DKD progression, including macroalbuminuria^7^ and the severity of glomerular and interstitial lesions.^8^, ^9^

Emerging experimental and clinical evidence reported the crucial role of renal inflammation in the pathogenesis and progression of DKD.^10^, ^11^, ^12^ The complement system, as an essential component of human immune system, was proved to participate in the development of DKD.^13^, ^14^, ^15^, ^16^, ^17^, ^18^ Targeting complement overactivation can improve renal function and ameliorate renal fibrosis in animal models of DKD.^19^, ^20^, ^21^

C3 is the central component of the complement system. All the three complement pathways, that is, the mannose‐binding lectin (MBL) pathway, the classic pathway, and the alternative pathway,^22^ converge at the formation of C3 convertase, which initiates the cleavage of C3 leading to the generation of C3a and C3b.^23^ C3b is further degraded to iC3b, C3c, and C3d.^24^ C1q binding with immune complex is the initial step of the classic pathway. Our previous study proved that DKD patients with renal C3c or C1q deposition were associated with more severe renal lesions.^25^ Therefore, the present study aimed to further investigate the association between clinicopathological parameters and renal outcomes in DKD patients with T2DM, especially the predictive value of complements deposition.

## 2 MATERIALS AND METHODS

### 2.1 Subjects

**FIGURE 1:**
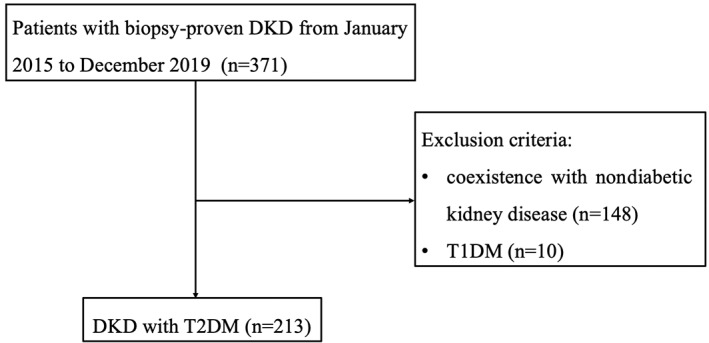
Flow chart of patient recruitment. DKD, diabetic kidney disease; T1DM, type 1 diabetes mellitus; T2DM, type 2 diabetes mellitus

A total of 371 patients with biopsy‐proven DKD at Renal Division of Peking University First Hospital between 1 January 2015 and 31 December 2019 were retrospectively recruited in this study. All patients met the criteria for the diagnosis of DM proposed by the American Diabetes Association.^26^ The pathological diagnosis of DKD was confirmed by renal pathologists according to the criteria proposed by the Renal Pathology Society in 2010.^27^ A total of 148 patients with concomitant nondiabetic kidney disease were excluded. Ten patients with type 1 diabetes mellitus were not enrolled. Finally, 213 patients with T2DM were included in analysis (Figure 1). The investigation was conducted according to the Declaration of Helsinki and was approved by the Ethics Committee of Peking University First Hospital (2017‐1280). Written informed consent was obtained from each participant.

### 2.2 Clinical data

Clinical data were collected at the time of renal biopsy, including sex, age, duration of diabetes, presence of diabetic retinopathy (DR), 24‐h proteinuria, hematuria (defined as ≥5 RBC/HP on urinary sediment), serum creatinine, hemoglobin A1c (HbA1c), triglyceride, low‐density lipoprotein, high‐density lipoprotein, erythrocyte sedimentation rate, C‐reactive protein, and serum C3. Estimated glomerular filtration rate (eGFR) was calculated by the Chronic Kidney Disease Epidemiology Collaboration equation.^28^

### 2.3 Renal histopathology

Light microscopy, direct immunofluorescence, and electron microscopy were routinely performed for each renal biopsy specimen. The biopsy specimens were independently reviewed and scored by two experienced renal pathologists. Light microscopic sections were stained with hematoxylin eosin, periodic acid‐Schiff, Masson trichrome, and periodic acid methenamine silver stain. Glomerular lesions were classified into class I, II, III, or IV based on glomerular basement membrane thickening, degree of mesangial expansion, presence of nodular sclerosis (Kimmelstiel‐Wilson lesions), and advanced diabetic glomerulosclerosis. On the basis of the affected proportion of the tubulointerstitial compartment, interstitial fibrosis and tubular atrophy (IFTA) were semiquantitatively scored as 0, 1, 2, or 3 (0, absent; 1, <25%; 2, 25–50%; 3, >50%). Interstitial inflammation was graded according to the infiltrated area (0, absent; 1, infiltration only in areas related to IFTA; 2, infiltration in areas without IFTA). Vascular changes were evaluated based on arteriolar hyalinosis and large vessel arteriosclerosis (0, absent; 1, at least one arteriolar hyalinosis or large vessel arteriosclerosis is present). The stainings of IgG, IgA, IgM, C3c, C1q, fibrinogen‐fibrin related antigen, and albumin were performed on frozen tissue using specific fluorescein‐conjugated antibodies and were evaluated under a fluorescence microscope. The intensity of staining was semiquantitatively graded on a scale of 0–4+. Repeated review of any scoring difference between the two pathologists was performed until a consensus was achieved. Electron microscopy was performed to exclude some renal morphologic lesions reminiscent of DKD,^29^ such as light‐chain deposition disease, amyloidosis, and membranoproliferative glomerulonephritis.

### 2.4 Outcomes

The primary end point was the progression to ESRD, which was defined as a requirement of permanent renal replacement therapies for >3 months. Participants were followed until the presence of ESRD or the end of the research (28 February 2021).

### 2.5 Statistical analysis

For continuous variables, data with normal distribution were expressed as mean ± SD and data with non‐normal distribution were expressed as median (interquartile range). Categorical variables were expressed as counts and percentages. Kaplan‐Meier analysis (log‐rank test) and univariate Cox regression were performed to explore potential predictors of renal outcomes. Candidate variables (*p* < .05) were further investigated with stepwise multivariate Cox regression analysis. Results were expressed as hazard ratios (HRs) and 95% confidence intervals (95% CIs). *p* values less than .05 were considered statistically significant. All statistical analyses were performed with SPSS 24.0.

## 3 RESULTS

### 3.1 General data

Among the 213 DKD patients, 163 were male and 50 were female, with an average age of 51.3 ± 11.6 years at the time of renal biopsy. The median duration of DM was 120 (60, 186) months. The median level of urinary protein was 3.8 (2.0, 7.3) g/24 h and 122/213 (57.3%) patients had hematuria. The serum albumin was 33.4 ± 6.1 g/L. The median serum creatinine was 180.5 (108.9, 310.4) μmol/L and eGFR was 35.6 (19.2, 60.3) mL/min/1.73m^2^. Of 213 patients, 160 (75.1%) showed renal insufficiency (defined as eGFR <60 mL/min/1.73m^2^) at renal biopsy. Of 213 patients, 148 (69.5%) had DR and the HbA1c was 7.1 ± 1.7% (Table 1).

**TABLE 1:** General data of DKD patients

Variables	Total patients (*n* = 213)
Age, years	51.3 ± 11.6
Male, *n* (%)	163 (76.5)
Duration of DM, months	120 (60, 186)
Proteinuria, g/24 h (0–0.15)	3.8 (2.0, 7.3)
Hematuria, *n* (%)	122 (57.3)
Serum albumin, g/L (40–55)	33.4 ± 6.1
Serum creatinine, μmol/L (44–133)	180.5 (108.9, 310.4)
eGFR, mL/min/1.73 m^2^	35.6 (19.2, 60.3)
Renal insufficiency, *n* (%)	160 (75.1)
HbA1c, % (4–6)	7.1 ± 1.7
DR, *n* (%)	148 (69.5)
TG, mmol/L (3.4–5.2)	1.77 (1.27, 2.74)
LDL, mmol/L (2.1–3.1)	2.89 ± 1.17
HDL, mmol/L (0.9–1.4)	0.98 ± 0.28
ESR, mm/1 h (0–15)	50.3 ± 31.7
CRP, mg/L (0–8)	1.76 (0.78, 4.09)
Serum C3, g/L (0.6–1.5)	0.89 ± 0.17

Abbreviations: CRP, C‐reactive protein; DKD, diabetic kidney disease; DM, diabetes mellitus; DR, diabetic retinopathy; eGFR, estimated glomerular filtration rate; ESR, erythrocyte sedimentation rate; HbA1c, hemoglobin A1c; HDL, high‐density lipoprotein; LDL, low‐density lipoprotein; TG, triglyceride.

### 3.2 Pathological characteristics

The detailed pathological characteristics of these DKD patients are presented in Table 2. Light microscopic sections had average 32.3 ± 14.2 glomeruli. Eight out of 213 (3.8%), 57/213 (26.8%), 122/213 (57.3%), and 26/213 (12.2%) patients were categorized as class I, II, III, and IV of glomerular lesions, respectively. IFTA scores of 1, 2, and 3 were had by 61/213 (28.6%), 83/213 (39.0%) and 69/213 (32.4%) patients, respectively. For interstitial inflammation, 82/213 (38.5%) patients were scored 1 and 131/213 (61.5%) patients were scored 2. IgG, IgM, and IgA deposition was observed in 174/213 (81.7%), 129/213 (60.6%), and 57/213 (26.8%) patients, respectively. As shown in Table S1, IgG deposition was mainly found in glomerular capillary walls (GCW) (164/174, 94.3%), tubular basement membrane (TBM) (155/174, 89.1%), and Bowman's capsule (60/174, 34.5%). IgM deposition was mainly found in GCW (61/129, 47.3%) and mesangium (97/129, 75.2%), as IgA deposition was mainly found in GCW and mesangium in 34/57 and 31/57 patients, respectively. C3c and C1q deposition was found in 133/213 (62.4%) and 45/213 (21.1%) patients, respectively. For C3c deposition in DKD patients, 54/133 (40.6%) were observed in GCW and 98/133 (73.7%) were observed in mesangium. For C1q deposition, 22/45 (48.9%) and 34/45 (75.6%) were detected in GCW and mesangium, respectively. A total of 172/213 (80.8%) patients had albumin deposition, which was mainly found in GCW (162/172, 94.2%), TBM (160/172, 93.0%), and Bowman's capsule (64/172, 37.2%). With electron microscopy, little, if any, electron‐dense deposit was observed in each specimen.

**TABLE 2:** Pathological characteristics of DKD patients

	Total patients (*n* = 213)
Light microscopy	
Glomerular class, *n* (%)	
class I/class II/class III/class IV	8 (3.8)/57 (26.8)/122 (57.3) /26 (12.2)
IFTA, *n* (%)	
0/1/2/3	0 (0)/61 (28.6)/83 (39.0)/69 (32.4)
Interstitial inflammation, *n* (%)	
0/1/2	0 (0)/82 (38.5)/131 (61.5)
Vascular lesions	
0/1	1 (0.5)/212 (99.5)
Immunofluorescence	
IgG deposition, *n* (%)	174 (81.7)
Scale 0/1+/2+/3+/4+	39/88/70/16/0
IgM deposition, *n* (%)	129 (60.6)
Scale 0/1+/2+/3+/4+	84/46/50/32/1
IgA deposition, *n* (%)	57 (26.8)
Scale 0/1+/2+/3+/4+	156/50/7/0/0
C3c deposition, *n* (%)	133 (62.4)
Scale 0/1+/2+/3+/4+	80/45/37/47/4
C1q deposition, *n* (%)	45 (21.1)
Scale 0/1+/2+/3+/4+	168/24/13/8/0
Albumin deposition, *n* (%)	172 (80.8)
Scale 0/1+/2+/3+/4+	41/78/75/19/0

Abbreviations: DKD, diabetic kidney disease; IFTA, interstitial fibrosis and tubular atrophy.

### 3.3 Risk factors for renal survival

During a median follow‐up of 23.0 (12.0, 39.0) months, 100/213 (46.9%) patients progressed to ESRD. The estimated median renal survival time was 39.0 months.

**FIGURE 2:**
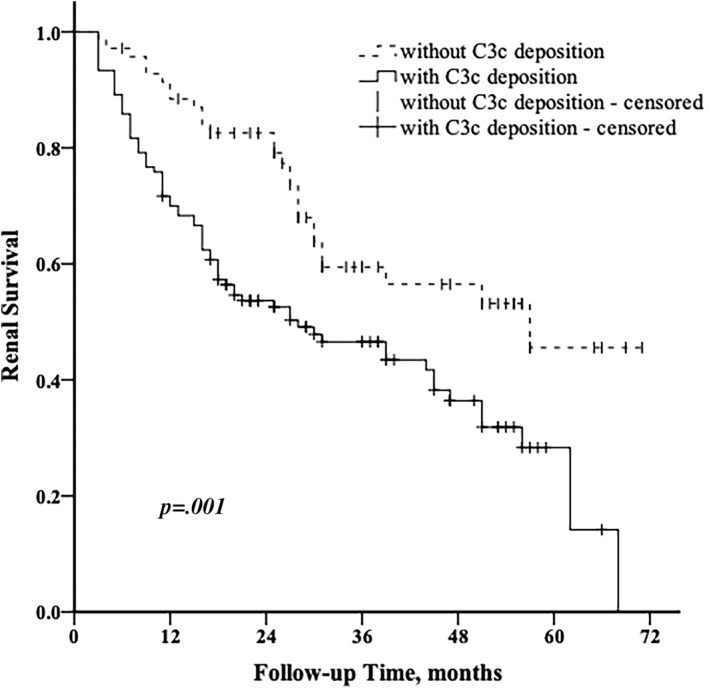
Kaplan–Meier survival curve in DKD patients with or without C3c deposition. DKD, diabetic kidney disease

In univariate analysis (Table 3), proteinuria (per 1 g/24 h increase, HR 1.171, 95% CI [1.126, 1.218], *p* < .001), eGFR < 60 mL/min/1.73m^2^ (HR 5.569, 95% CI [2.795, 11.096], *p* < .001), coexistence with DR (HR 2.280, 95% CI [1.380, 3.766], *p* = .001), advanced glomerular lesions (Class III and IV vs. I and II; HR 3.196, 95% CI [1.843, 5.543], *p* < .001), higher IFTA score (Score 2 and 3 vs. 0 and 1; HR 4.906, 95% CI [2.676, 8.994], *p* < 0.001), higher grade of interstitial inflammation (Score 2 vs. 0 and 1; HR 2.894, 95% CI [1.823, 4.595], *p* < .001), higher intensity of C3c deposition (per 1+ increase, HR 1.452, 95% CI [1.234, 1.708], *p* < .001), and higher intensity of C1q deposition (per 1+ increase, HR 1.417, 95% CI [1.156, 1.738], *p* = .001) were potential risk factors for progressing to ESRD in DKD patients. Kaplan‐Meier analysis showed that patients with C3c deposition had worse renal outcomes compared with those without C3c deposition (log‐rank test, *p* = .001, Figure 2), as did C1q deposition (log‐rank test, *p* < .001).

**TABLE 3:** Potential prognostic factors for renal outcomes determined by univariate and multivariate Cox regression analysis in DKD patients

Variable	Univariate analysis		Multivariate analysis	
	HR (95% CI)	*p* value	HR (95% CI)	*p* value
sex (male)	1.161 (0.722, 1.865)	.538		
Age ≥ 60 years (yes vs. no)	1.059 (0.652, 1.720)	.816		
Proteinuria (g/24 h)	1.171 (1.126, 1.218)	**<.001**	1.134 (1.079, 1.191)	**<.001**
Hematuria (yes vs. no)	1.508 (0.991, 2.295)	.055		
eGFR<60 mL/min/1.73m^2^ (yes vs. no)	5.569 (2.795, 11.096)	**<.001**		
HbA1c (%)	0.828 (0.707, 0.970)	**.019**		
DR (yes vs. no)	2.280 (1.380, 3.766)	**.001**		
TG, mmol/L	0.989 (0.869, 1.127)	.873		
LDL, mmol/L	1.208 (1.015, 1.438)	**.033**		
HDL, mmol/L	0.710 (0.347, 1.453)	.349		
ESR > 15 mm/1h (yes vs. no)	3.122 (1.433, 6.800)	**.004**		
CRP > 8 mg/L (yes vs. no)	1.327 (0.679, 2.592)	.408		
Serum C3 (g/L)	0.309 (0.091, 1.054)	.061		
Glomerular class (III and IV vs. I and II)	3.196 (1.843, 5.543)	**<.001**		
IFTA (score 2 and 3 vs. 0 and 1)	4.906 (2.676, 8.994)	**<.001**	3.925 (1.855, 8.304)	**<.001**
Interstitial inflammation (score 2 vs. 0 and 1)	2.894 (1.823, 4.595)	**<.001**		
IgG deposition (per +)	1.184 (0.937, 1.496)	.157		
IgM deposition (per +)	1.181 (0.990, 1.407)	.064		
IgA deposition (per +)	0.803 (0.529, 1.219)	.302		
C3c deposition (per +)	1.452 (1.234, 1.708)	**<.001**	1.299 (1.073, 1.573)	**.007**
C1q deposition (per +)	1.417 (1.156, 1.738)	**.001**		
Albumin deposition (per +)	1.218 (0.970, 1.528)	.089		

*Note:* Bold values indicates the p values less than .05 were considered statistically significant.

Abbreviations: CI, confidence interval; CRP, C‐reactive protein; DKD, diabetic kidney disease; DR, diabetic retinopathy; eGFR, estimated glomerular filtration rate; ESR, erythrocyte sedimentation rate; HbA1c, hemoglobin A1c; HDL, high‐density lipoprotein; HR, hazard ratio; IFTA, interstitial fibrosis and tubular atrophy; LDL, low‐density lipoprotein; TG, triglyceride.

Multivariate Cox regression analysis showed that proteinuria (per 1 g/24 h increase, HR 1.134, 95% CI [1.079, 1.191], *p* < .001), IFTA score (score 2 and 3 vs. 0 and 1, HR 3.925, 95% CI [1.855, 8.304], *p* < .001), and C3c deposition (per 1+ increase, HR 1.299, 95% CI [1.073, 1.573], *p* = .007) were independent risk factors for developing ESRD.

## 4 DISCUSSION

Although antidiabetic treatments have brought great improvement in various complications of diabetes, patients with DKD progressing to ESRD still increased rapidly.^30^ Noninvasive predictors for progression of DKD were eagerly needed. Increasing studies have confirmed that immune inflammation, especially innate immunity, plays a vital role in the pathogenesis of DKD, including tumor necrosis factor receptor superfamily members, interleukin‐8‐CXCR1/2 axis and immune‐related molecule B7‐1.^10^, ^11^, ^12^ As a central component of innate immunity, the complement overactivation participates in DKD progression.^13^, ^14^, ^15^, ^16^, ^17^, ^18^

In our previous study, it was found that patients with C3c deposition in the kidney had more severe renal injury, including lower eGFR, and higher scores for IFTA.^25^ In the current study, we explored the association between clinicopathological parameters and renal outcomes in DKD patients with T2DM, and we found that after adjusting various factors, C3c deposition was an independent predictor for ESRD.

Renal complements depositions were not rare in patients with DKD. In the current study, C3c deposition was observed in 62.4% of the patients. C3c was a downstream product of C3 in the complement system. Transcriptome analysis showed a 6‐fold increase in gene expression of C3 in glomeruli tissue from DKD patients compared with healthy people.^31^ Upregulated expression of C3 was detected in the kidney of OVE26 diabetic mouse and was associated with severe albuminuria.^32^ Our previous study in mice showed that C3a receptor deficiency could attenuate diabetic kidney disease through suppressing inflammatory responses and T‐cell adaptive immunity.^33^ C3a receptor antagonists were proved to ameliorate endothelial‐myofibroblast transition and alleviate fibrosis of the kidney in streptozotocin‐induced DKD rat.^20^ Taken together, C3 and its activated fragments are promising therapeutic targets for DKD.

In the current study, higher intensity of C1q deposition was associated with poor renal survival in the univariate analysis. However, after adjusting for various factors including C3c deposition, the predictive value of C1q deposition was diminished. Because C1q is the key component of the classic pathway, the results indicated that the MBL pathway and alternative pathway might play more important roles in the pathogenesis of DKD, as compared with the classic pathway. The overactivated MBL pathway and/or alternative pathway would lead to the formation of C3 convertase, which might be the upstream pathway of C3 participating in progression of DKD.

The study was conducted in a single center, and multicentral studies with larger sample size are needed.

In conclusion, C3c deposition was an independent predictor for ESRD in DKD patients with T2DM. It might provide a biomarker indicating DKD progression and potential therapeutic target.

## CONFLICT OF INTEREST

The authors have no conflicts of interest to declare.

## Supporting information


**Table S1** The deposition pattern of immunofluorescent staining in DKD patients.
**Figure S1**. Venn diagram of double staining of C3c with IgG or IgM.
