# Supplementary material for: C3c deposition predicts worse renal outcomes in patients with biopsy‐proven diabetic kidney disease in type 2 diabetes mellitus
Source: J Diabetes. 2022 Mar 24;14(4):291–7. doi: 10.1111/1753-0407.13264 (PMC9060041; doi:10.1111/1753-0407.13264)
Supplement: Supplementary file 1 — Table S1 The deposition pattern of immunofluorescent staining in DKD patients. Figure S1. Venn diagram of double staining of C3c with IgG or IgM. [file JDB-14-291-s001.docx]

Supplemental Table 1. The deposition pattern of immunofluorescent staining in DKD patients.

|  | positive  total, n | GCW,  n (%) | TBM,  n (%) | mesangium,  n (%) | Bowman’s capsule, n (%) | sclerosis glomeruli, n (%) |
| --- | --- | --- | --- | --- | --- | --- |
| IgG | 174 | 164 (94.3) | 155 (89.1) | 13 (7.5) | 60 (34.5) | 4 (2.3) |
| IgM | 129 | 61(47.3) | 17 (13.2) | 97 (75.2) | 8 (6.2) | 10 (7.8) |
| IgA | 57 | 34 (59.6) | 23 (40.4) | 31 (54.4) | 6 (10.5) | 1 (1.8) |
| C3c | 133 | 54 (40.6) | 19 (14.3) | 98(73.7) | 13 (9.8) | 21 (15.8) |
| C1q | 45 | 22 (48.9) | 5 (11.1) | 34 (75.6) | 3 (6.7) | 5 (11.1) |
| albumin | 172 | 162 (94.2) | 160 (93.0) | 7 (4.1) | 64 (37.2) | 1 (0.6) |
| C3c+IgG+ | 54 | 42 (77.8) | 16 (29.6) | 13 (24.1) | 8 (14.8) | 5 (9.3) |
| C3c+IgM+ | 88 | 40 (45.5) | 8 (9.1) | 69 (78.4) | 2 (2.3) | 9 (10.2) |
| C3c+C1q+ | 36 | 19 (52.8) | 2 (5.6) | 28 (77.8) | 0 (0) | 6 (16.7) |

C3c+C1q+ refers cases with simultaneous staining of C3c and C1q in the same location; C3c+IgG+ refers cases with simultaneous staining of C3c and IgG in the same location; C3c+IgM+ refers cases with simultaneous staining of C3c and IgM in the same location; GCW, glomerular capillary walls; TBM, tubular basement membrane.


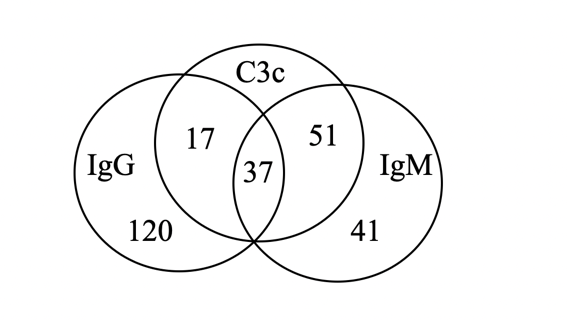


Supplemental Figure 1. Venn diagram of double staining of C3c with IgG or IgM.
